# Tetra­kis(ethyl pyridine-4-carboxyl­ate-κ*N*)bis­(thio­cyanato-κ*N*)cobalt(II)

**DOI:** 10.1107/S1600536812021988

**Published:** 2012-05-19

**Authors:** Xiu-Ling Feng, Yu-Ping Zhang

**Affiliations:** a College of Chemistry and Chemical Engineering, Huaihua University, Huaihua 418008, People’s Republic of China; b Wuling Electric Power Group Corporation, Changsha 410000, People’s Republic of China

## Abstract

In the title complex, [Co(NCS)_2_(C_8_H_9_NO_2_)_4_], the Co^II^ atom is six-coordinated by four N atoms from four ethyl pyridine-4-carboxyl­ate ligands in the equatorial plane and two N atoms of thio­cyanate ligands in the axial positions, showing a slightly distorted octa­hedral geometry. The structure exhibits disorder in one of the ethyl chains, which was refined using a two-site model with 0.70 (6):0.30 (6) occupancy.

## Related literature

For the structures of related complexes containing ethyl pyridine-4-carboxyl­ate ligands, see: Wang *et al.* (2012 ▶). For other related structures, see: Manna *et al.* (2008 ▶); Diehr *et al.* (2011 ▶).
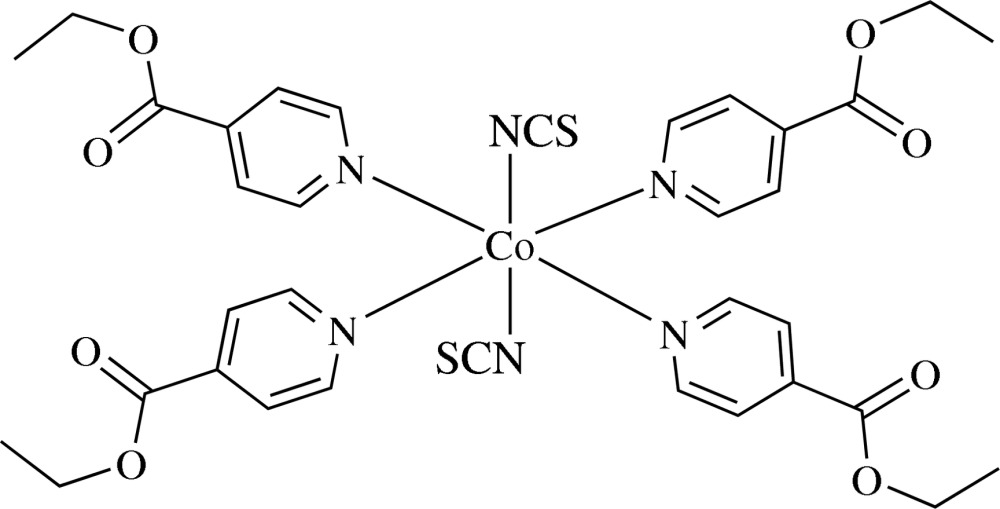


## Experimental

### 

#### Crystal data

[Co(NCS)_2_(C_8_H_9_NO_2_)_4_]
*M* *_r_* = 779.74
Monoclinic, 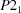

*a* = 11.2190 (11) Å
*b* = 14.3742 (16) Å
*c* = 12.0189 (13) Å
β = 96.430 (1)°
*V* = 1926.0 (4) Å^3^
*Z* = 2
Mo *K*α radiation
μ = 0.61 mm^−1^
*T* = 298 K
0.48 × 0.40 × 0.30 mm

#### Data collection

Bruker SMART 1000 CCD diffractometer
Absorption correction: multi-scan (*SADABS*; Bruker, 2002 ▶) *T*_min_ = 0.759, *T*_max_ = 0.838
10061 measured reflections
5985 independent reflections
4025 reflections with *I* > 2σ(*I*)
*R*_int_ = 0.037

#### Refinement

*R*[*F* ^2^ > 2σ(*F* ^2^)] = 0.047
*wR*(*F* ^2^) = 0.122
*S* = 1.03
5985 reflections
484 parameters
1 restraint
H-atom parameters constrained
Δρ_max_ = 0.37 e Å^−3^
Δρ_min_ = −0.28 e Å^−3^
Absolute structure: Flack (1983 ▶), 2433 Friedel pairs
Flack parameter: −0.04 (2)

### 

Data collection: *SMART* (Bruker, 2002 ▶); cell refinement: *SAINT* (Bruker, 2002 ▶); data reduction: *SAINT*; program(s) used to solve structure: *SHELXS97* (Sheldrick, 2008 ▶); program(s) used to refine structure: *SHELXL97* (Sheldrick, 2008 ▶); molecular graphics: *SHELXTL* (Sheldrick, 2008 ▶); software used to prepare material for publication: *SHELXTL*.

## Supplementary Material

Crystal structure: contains datablock(s) I, global. DOI: 10.1107/S1600536812021988/ez2290sup1.cif

Structure factors: contains datablock(s) I. DOI: 10.1107/S1600536812021988/ez2290Isup2.hkl

Additional supplementary materials:  crystallographic information; 3D view; checkCIF report

## Figures and Tables

**Fig. 1.**
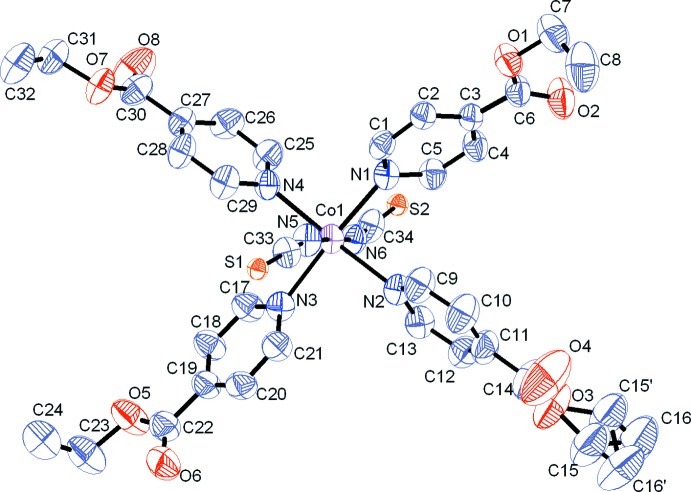
The molecular structure of the title molecule with the atom-numbering scheme. Displacement ellipsoids are drawn at the 50% probability level and H atoms have been omitted for clarity. Both disorder models are shown.

## supplementary crystallographic information

### Comment

Coordination compounds with N-heterocyclic ligands have been extensively studied due to the diversity of their structures. Many coordination complexes with N-heterocyclic and thiocyanate ligands have been reported (Wang *et al.*, 2012; Manna *et al.*, 2008; Diehr *et al.*, 2011). As part of our further studies on this class of compounds, we report here the crystal structure of the title compound.

In the title complex, Co(C_8_H_9_NO_2_)_4_(SCN)_2_, the Co^II^ atom is six-coordinated with four N atoms from four ethyl pyridine-4-carboxylate ligands in the equatorial plane and two N atoms of thiocyanatos in the axial positions(Fig.1), showing a slightly distorted octahedral geometry. The equatorial Co—N distances are in the range of 2.192 (4) ~ 2.209 (4) Å which are significantly longer than the axial Co—N bond distances of 2.041 (5) and 2.071 (5) Å. The title complex exhibits a zero-dimensional structure.

### Experimental

A mixture of CoCl_2_.6H_2_O (0.5 mmol), pyridine-4-carboxylic acid (2 mmol), 3-aminopropanoic acid (1 mmol) and KSCN (1 mmol) in a 40% aqueous solution of ethanol (15 mL) was heated at 413 K in a Teflon-lined stainless steel autoclave for three days. The reaction system was then slowly cooled to room temperature. Red crystals of the title compound suitable for single-crystal X-ray diffraction analysis were obtained by slow evaporation of aqua solution over a period of one month (yield 30% based on Co).

### Refinement

All H atoms attached to C atoms were fixed geometrically and treated as riding with C—H = 0.93 Å (CH), 0.97 Å (CH_2_) and 0.96 Å (CH_3_), and with *U*iso(H) = 1.5Ueq(C) for the methyl group and *U*iso(H) = 1.2Ueq(C) for all others. The two C atoms (C15 and C16) of an ethyl pyridine-4-carboxylate are disordered over two positions in a 0.70 (6):0.30 (6) ratio.

### Crystal data

**Table:**

[Co(NCS)_2_(C_8_H_9_NO_2_)_4_]	*F*(000) = 810
*M**_r_* = 779.74	*D*_x_ = 1.345 Mg m^−^^3^
Monoclinic, *P*2_1_	Mo *K*α radiation, λ = 0.71073 Å
Hall symbol: P 2yb	Cell parameters from 2899 reflections
*a* = 11.2190 (11) Å	θ = 2.3–21.7°
*b* = 14.3742 (16) Å	µ = 0.61 mm^−^^1^
*c* = 12.0189 (13) Å	*T* = 298 K
β = 96.430 (1)°	Block, red
*V* = 1926.0 (4) Å^3^	0.48 × 0.40 × 0.30 mm
*Z* = 2	

### Data collection

**Table:**

Bruker SMART 1000 CCD diffractometer	5985 independent reflections
Radiation source: fine-focus sealed tube	4025 reflections with *I* > 2σ(*I*)
Graphite monochromator	*R*_int_ = 0.037
phi and ω scans	θ_max_ = 25.0°, θ_min_ = 2.3°
Absorption correction: multi-scan (*SADABS*; Bruker, 2002)	*h* = −13→13
*T*_min_ = 0.759, *T*_max_ = 0.838	*k* = −17→16
10061 measured reflections	*l* = −14→13

### Refinement

**Table:**

Refinement on *F*^2^	Secondary atom site location: difference Fourier map
Least-squares matrix: full	Hydrogen site location: inferred from neighbouring sites
*R*[*F*^2^ > 2σ(*F*^2^)] = 0.047	H-atom parameters constrained
*wR*(*F*^2^) = 0.122	*w* = 1/[σ^2^(*F*_o_^2^) + (0.0362*P*)^2^ + 0.8634*P*] where *P* = (*F*_o_^2^ + 2*F*_c_^2^)/3
*S* = 1.03	(Δ/σ)_max_ = 0.001
5985 reflections	Δρ_max_ = 0.37 e Å^−^^3^
484 parameters	Δρ_min_ = −0.28 e Å^−^^3^
1 restraint	Absolute structure: Flack (1983), 2433 Friedel pairs
Primary atom site location: structure-invariant direct methods	Flack parameter: −0.04 (2)

### Special details

**Table:**

Geometry. All e.s.d.'s (except the e.s.d. in the dihedral angle between two l.s. planes) are estimated using the full covariance matrix. The cell e.s.d.'s are taken into account individually in the estimation of e.s.d.'s in distances, angles and torsion angles; correlations between e.s.d.'s in cell parameters are only used when they are defined by crystal symmetry. An approximate (isotropic) treatment of cell e.s.d.'s is used for estimating e.s.d.'s involving l.s. planes.
Refinement. Refinement of *F*^2^ against ALL reflections. The weighted *R*-factor *wR* and goodness of fit *S* are based on *F*^2^, conventional *R*-factors *R* are based on *F*, with *F* set to zero for negative *F*^2^. The threshold expression of *F*^2^ > σ(*F*^2^) is used only for calculating *R*-factors(gt) *etc*. and is not relevant to the choice of reflections for refinement. *R*-factors based on *F*^2^ are statistically about twice as large as those based on *F*, and *R*- factors based on ALL data will be even larger.

### Fractional atomic coordinates and isotropic or equivalent isotropic displacement parameters (Å^2^)

**Table:**

	*x*	*y*	*z*	*U*_iso_*/*U*_eq_	Occ. (<1)
Co1	0.41695 (6)	0.42870 (5)	0.29066 (6)	0.0488 (2)	
S1	0.5628 (2)	0.74028 (15)	0.3384 (2)	0.1067 (8)	
S2	0.2556 (2)	0.12627 (16)	0.2389 (3)	0.1262 (9)	
N1	0.4685 (4)	0.3954 (3)	0.1232 (4)	0.0513 (12)	
N2	0.5961 (4)	0.3757 (3)	0.3506 (4)	0.0532 (12)	
N3	0.3800 (4)	0.4617 (3)	0.4615 (4)	0.0532 (12)	
N4	0.2373 (4)	0.4792 (3)	0.2233 (4)	0.0497 (11)	
N5	0.4864 (4)	0.5617 (4)	0.2819 (4)	0.0579 (12)	
N6	0.3460 (4)	0.2991 (4)	0.3050 (4)	0.0549 (12)	
O1	0.6918 (3)	0.4031 (3)	−0.2104 (3)	0.0649 (11)	
O2	0.7291 (4)	0.2603 (3)	−0.1435 (4)	0.0874 (14)	
O3	0.9606 (5)	0.2026 (6)	0.4967 (6)	0.131 (3)	
O4	1.0350 (5)	0.3214 (6)	0.4168 (7)	0.168 (3)	
O5	0.1750 (4)	0.5513 (4)	0.7882 (4)	0.0924 (15)	
O6	0.3614 (4)	0.5993 (4)	0.8411 (4)	0.0932 (16)	
O7	−0.1268 (4)	0.6682 (4)	0.1195 (4)	0.0735 (13)	
O8	−0.1984 (4)	0.5241 (4)	0.0914 (5)	0.1110 (19)	
C1	0.4928 (5)	0.4607 (4)	0.0515 (5)	0.0521 (14)	
H1	0.4649	0.5206	0.0620	0.063*	
C2	0.5579 (4)	0.4448 (4)	−0.0386 (4)	0.0516 (14)	
H2	0.5724	0.4927	−0.0873	0.062*	
C3	0.6001 (5)	0.3566 (4)	−0.0539 (4)	0.0485 (13)	
C4	0.5708 (5)	0.2873 (4)	0.0171 (5)	0.0566 (15)	
H4	0.5942	0.2262	0.0064	0.068*	
C5	0.5065 (5)	0.3098 (4)	0.1042 (5)	0.0558 (14)	
H5	0.4885	0.2625	0.1525	0.067*	
C6	0.6784 (5)	0.3337 (5)	−0.1399 (5)	0.0598 (15)	
C7	0.7686 (6)	0.3856 (5)	−0.2978 (6)	0.086 (2)	
H7A	0.7466	0.4275	−0.3600	0.103*	
H7B	0.7561	0.3225	−0.3250	0.103*	
C8	0.8956 (7)	0.3986 (6)	−0.2577 (7)	0.117 (3)	
H8A	0.9081	0.4608	−0.2296	0.176*	
H8B	0.9431	0.3887	−0.3183	0.176*	
H8C	0.9188	0.3549	−0.1989	0.176*	
C9	0.6916 (5)	0.4149 (5)	0.3136 (5)	0.0694 (17)	
H9	0.6804	0.4659	0.2660	0.083*	
C10	0.8057 (6)	0.3830 (5)	0.3430 (6)	0.084 (2)	
H10	0.8707	0.4133	0.3174	0.101*	
C11	0.8235 (5)	0.3071 (5)	0.4097 (6)	0.0703 (18)	
C12	0.7267 (6)	0.2636 (5)	0.4472 (5)	0.0688 (17)	
H12	0.7365	0.2110	0.4924	0.083*	
C13	0.6137 (5)	0.3007 (5)	0.4153 (5)	0.0607 (15)	
H13	0.5474	0.2719	0.4403	0.073*	
C14	0.9529 (8)	0.2781 (8)	0.4441 (8)	0.101 (3)	
C15	1.087 (2)	0.186 (2)	0.546 (3)	0.130 (9)	0.70 (6)
H15A	1.0961	0.1992	0.6258	0.157*	0.70 (6)
H15B	1.1421	0.2239	0.5099	0.157*	0.70 (6)
C16	1.108 (3)	0.084 (3)	0.526 (3)	0.166 (12)	0.70 (6)
H16A	1.0604	0.0470	0.5706	0.249*	0.70 (6)
H16B	1.1914	0.0697	0.5454	0.249*	0.70 (6)
H16C	1.0861	0.0699	0.4480	0.249*	0.70 (6)
C15'	1.056 (5)	0.136 (5)	0.483 (7)	0.12 (2)	0.30 (6)
H15C	1.0854	0.1455	0.4109	0.149*	0.30 (6)
H15D	1.0256	0.0733	0.4857	0.149*	0.30 (6)
C16'	1.158 (6)	0.150 (5)	0.576 (5)	0.14 (2)	0.30 (6)
H16D	1.1395	0.2019	0.6219	0.212*	0.30 (6)
H16E	1.2308	0.1628	0.5440	0.212*	0.30 (6)
H16F	1.1674	0.0951	0.6211	0.212*	0.30 (6)
C17	0.2728 (5)	0.4426 (5)	0.4944 (4)	0.0608 (15)	
H17	0.2186	0.4074	0.4476	0.073*	
C18	0.2393 (5)	0.4734 (4)	0.5958 (5)	0.0634 (17)	
H18	0.1632	0.4607	0.6157	0.076*	
C19	0.3204 (5)	0.5229 (4)	0.6662 (4)	0.0590 (15)	
C20	0.4324 (5)	0.5384 (4)	0.6346 (5)	0.0609 (16)	
H20	0.4902	0.5694	0.6824	0.073*	
C21	0.4582 (5)	0.5081 (4)	0.5332 (5)	0.0605 (16)	
H21	0.5341	0.5202	0.5125	0.073*	
C22	0.2895 (6)	0.5621 (5)	0.7750 (5)	0.0677 (16)	
C23	0.1327 (8)	0.5898 (6)	0.8912 (6)	0.101 (3)	
H23A	0.0654	0.5533	0.9108	0.121*	
H23B	0.1965	0.5855	0.9525	0.121*	
C24	0.0957 (9)	0.6875 (8)	0.8753 (7)	0.121 (3)	
H24A	0.1641	0.7247	0.8629	0.182*	
H24B	0.0629	0.7093	0.9410	0.182*	
H24C	0.0361	0.6923	0.8118	0.182*	
C25	0.1515 (5)	0.4226 (5)	0.1774 (4)	0.0635 (14)	
H25	0.1700	0.3600	0.1699	0.076*	
C26	0.0371 (5)	0.4514 (4)	0.1404 (5)	0.0652 (17)	
H26	−0.0193	0.4093	0.1078	0.078*	
C27	0.0068 (5)	0.5447 (4)	0.1526 (5)	0.0515 (14)	
C28	0.0947 (5)	0.6025 (4)	0.1971 (5)	0.0624 (16)	
H28	0.0793	0.6657	0.2042	0.075*	
C29	0.2082 (5)	0.5672 (4)	0.2323 (5)	0.0607 (15)	
H29	0.2666	0.6082	0.2638	0.073*	
C30	−0.1169 (6)	0.5775 (6)	0.1176 (5)	0.0671 (18)	
C31	−0.2449 (6)	0.7085 (6)	0.0900 (6)	0.087 (2)	
H31A	−0.3019	0.6828	0.1366	0.104*	
H31B	−0.2731	0.6957	0.0122	0.104*	
C32	−0.2313 (6)	0.8104 (6)	0.1087 (7)	0.105 (3)	
H32A	−0.2091	0.8222	0.1869	0.157*	
H32B	−0.3059	0.8409	0.0849	0.157*	
H32C	−0.1701	0.8339	0.0665	0.157*	
C33	0.5190 (5)	0.6359 (5)	0.3058 (5)	0.0550 (14)	
C34	0.3064 (5)	0.2267 (5)	0.2801 (5)	0.0580 (16)	

### Atomic displacement parameters (Å^2^)

**Table:**

	*U*^11^	*U*^22^	*U*^33^	*U*^12^	*U*^13^	*U*^23^
Co1	0.0440 (4)	0.0428 (4)	0.0603 (4)	−0.0030 (4)	0.0096 (3)	−0.0021 (4)
S1	0.1289 (18)	0.0693 (14)	0.1272 (18)	−0.0407 (13)	0.0373 (14)	−0.0361 (13)
S2	0.1069 (17)	0.0605 (13)	0.208 (3)	−0.0263 (13)	0.0041 (17)	−0.0220 (16)
N1	0.048 (3)	0.046 (3)	0.061 (3)	0.002 (2)	0.009 (2)	−0.001 (2)
N2	0.046 (3)	0.050 (3)	0.064 (3)	−0.005 (2)	0.010 (2)	0.001 (2)
N3	0.047 (3)	0.051 (3)	0.062 (3)	−0.006 (2)	0.005 (2)	−0.004 (2)
N4	0.049 (3)	0.040 (3)	0.061 (3)	−0.001 (2)	0.010 (2)	−0.002 (2)
N5	0.055 (3)	0.047 (3)	0.073 (3)	−0.007 (3)	0.013 (2)	0.000 (3)
N6	0.051 (3)	0.048 (3)	0.066 (3)	0.000 (3)	0.007 (2)	−0.001 (3)
O1	0.078 (3)	0.057 (3)	0.064 (2)	0.005 (2)	0.0280 (19)	−0.001 (2)
O2	0.108 (4)	0.062 (3)	0.100 (4)	0.026 (3)	0.045 (3)	0.006 (3)
O3	0.081 (4)	0.138 (6)	0.169 (7)	0.042 (4)	−0.005 (4)	0.028 (5)
O4	0.060 (4)	0.196 (8)	0.246 (8)	−0.006 (4)	0.006 (4)	0.077 (7)
O5	0.097 (4)	0.118 (4)	0.068 (3)	−0.019 (3)	0.031 (3)	−0.024 (3)
O6	0.096 (4)	0.101 (4)	0.080 (3)	0.010 (3)	−0.002 (3)	−0.036 (3)
O7	0.048 (3)	0.072 (3)	0.099 (4)	0.014 (2)	0.001 (2)	0.007 (3)
O8	0.059 (3)	0.106 (4)	0.160 (5)	−0.019 (3)	−0.022 (3)	0.014 (4)
C1	0.054 (3)	0.038 (3)	0.063 (4)	0.006 (3)	0.002 (3)	0.004 (3)
C2	0.054 (3)	0.049 (4)	0.052 (3)	0.006 (3)	0.007 (2)	0.001 (3)
C3	0.052 (3)	0.044 (3)	0.050 (3)	0.007 (3)	0.007 (3)	−0.001 (3)
C4	0.066 (4)	0.040 (3)	0.065 (4)	0.008 (3)	0.015 (3)	−0.003 (3)
C5	0.060 (3)	0.046 (4)	0.064 (4)	0.001 (3)	0.013 (3)	0.005 (3)
C6	0.065 (4)	0.049 (4)	0.067 (4)	0.011 (3)	0.014 (3)	−0.005 (3)
C7	0.102 (5)	0.084 (5)	0.080 (4)	0.003 (4)	0.041 (4)	−0.006 (4)
C8	0.095 (6)	0.111 (8)	0.157 (7)	0.007 (5)	0.062 (5)	0.001 (6)
C9	0.048 (3)	0.074 (5)	0.089 (4)	−0.004 (4)	0.015 (3)	0.010 (4)
C10	0.052 (4)	0.087 (5)	0.115 (6)	−0.003 (4)	0.015 (4)	0.012 (5)
C11	0.044 (4)	0.080 (5)	0.086 (5)	0.008 (4)	0.003 (3)	−0.011 (4)
C12	0.067 (4)	0.063 (4)	0.075 (4)	0.005 (3)	0.000 (3)	0.002 (3)
C13	0.051 (4)	0.060 (4)	0.072 (4)	−0.007 (3)	0.011 (3)	−0.004 (3)
C14	0.068 (6)	0.110 (8)	0.125 (7)	0.014 (5)	0.012 (5)	0.027 (6)
C15	0.084 (13)	0.14 (2)	0.160 (19)	0.025 (14)	−0.019 (14)	0.007 (16)
C16	0.118 (19)	0.15 (3)	0.22 (2)	0.044 (18)	−0.020 (18)	0.01 (2)
C15'	0.09 (3)	0.13 (5)	0.15 (4)	0.03 (3)	−0.01 (3)	0.01 (4)
C16'	0.10 (4)	0.13 (5)	0.18 (4)	0.01 (3)	−0.04 (3)	0.03 (4)
C17	0.056 (3)	0.068 (4)	0.059 (3)	−0.016 (3)	0.005 (2)	−0.011 (3)
C18	0.060 (4)	0.076 (5)	0.056 (4)	−0.013 (3)	0.015 (3)	−0.008 (3)
C19	0.067 (4)	0.058 (4)	0.051 (3)	0.001 (3)	0.005 (3)	−0.007 (3)
C20	0.056 (4)	0.066 (4)	0.058 (4)	−0.009 (3)	−0.004 (3)	−0.010 (3)
C21	0.051 (3)	0.068 (4)	0.062 (4)	−0.007 (3)	0.006 (3)	−0.006 (3)
C22	0.077 (5)	0.064 (4)	0.061 (4)	0.000 (4)	0.004 (4)	−0.009 (3)
C23	0.113 (6)	0.119 (7)	0.078 (5)	−0.014 (6)	0.038 (5)	−0.018 (5)
C24	0.136 (9)	0.137 (9)	0.092 (7)	0.025 (7)	0.018 (6)	−0.016 (6)
C25	0.060 (3)	0.049 (3)	0.079 (4)	−0.001 (4)	−0.005 (3)	−0.004 (4)
C26	0.060 (4)	0.057 (5)	0.075 (4)	−0.014 (3)	−0.011 (3)	−0.003 (3)
C27	0.043 (3)	0.053 (4)	0.060 (3)	−0.001 (3)	0.009 (3)	0.009 (3)
C28	0.046 (3)	0.049 (4)	0.091 (4)	−0.001 (3)	0.006 (3)	0.004 (3)
C29	0.048 (3)	0.049 (4)	0.085 (4)	−0.006 (3)	0.005 (3)	0.001 (3)
C30	0.049 (4)	0.073 (5)	0.077 (4)	−0.002 (4)	−0.002 (3)	0.011 (4)
C31	0.060 (4)	0.101 (7)	0.096 (5)	0.022 (4)	−0.005 (4)	0.000 (5)
C32	0.071 (5)	0.107 (7)	0.132 (7)	0.024 (5)	−0.005 (4)	0.012 (6)
C33	0.052 (3)	0.052 (4)	0.063 (4)	−0.006 (3)	0.016 (3)	0.000 (3)
C34	0.046 (3)	0.052 (4)	0.078 (4)	0.000 (3)	0.014 (3)	0.009 (3)

### Geometric parameters (Å, º)

**Table:**

Co1—N6	2.041 (5)	C10—H10	0.9300
Co1—N5	2.071 (5)	C11—C12	1.372 (8)
Co1—N3	2.192 (4)	C11—C14	1.523 (10)
Co1—N2	2.194 (5)	C12—C13	1.390 (8)
Co1—N1	2.208 (4)	C12—H12	0.9300
Co1—N4	2.209 (4)	C13—H13	0.9300
S1—C33	1.614 (7)	C15—C16	1.51 (7)
S2—C34	1.610 (7)	C15—H15A	0.9700
N1—C1	1.322 (6)	C15—H15B	0.9700
N1—C5	1.331 (6)	C16—H16A	0.9600
N2—C13	1.330 (7)	C16—H16B	0.9600
N2—C9	1.331 (7)	C16—H16C	0.9600
N3—C17	1.336 (6)	C15'—C16'	1.52 (14)
N3—C21	1.338 (6)	C15'—H15C	0.9700
N4—C29	1.314 (7)	C15'—H15D	0.9700
N4—C25	1.333 (7)	C16'—H16D	0.9600
N5—C33	1.153 (7)	C16'—H16E	0.9600
N6—C34	1.157 (7)	C16'—H16F	0.9600
O1—C6	1.329 (7)	C17—C18	1.387 (7)
O1—C7	1.454 (7)	C17—H17	0.9300
O2—C6	1.201 (7)	C18—C19	1.370 (8)
O3—C14	1.254 (10)	C18—H18	0.9300
O3—C15'	1.46 (6)	C19—C20	1.370 (8)
O3—C15	1.49 (3)	C19—C22	1.500 (8)
O4—C14	1.188 (9)	C20—C21	1.356 (7)
O5—C22	1.321 (7)	C20—H20	0.9300
O5—C23	1.482 (8)	C21—H21	0.9300
O6—C22	1.193 (7)	C23—C24	1.471 (12)
O7—C30	1.308 (8)	C23—H23A	0.9700
O7—C31	1.453 (7)	C23—H23B	0.9700
O8—C30	1.209 (8)	C24—H24A	0.9600
C1—C2	1.391 (7)	C24—H24B	0.9600
C1—H1	0.9300	C24—H24C	0.9600
C2—C3	1.373 (7)	C25—C26	1.374 (7)
C2—H2	0.9300	C25—H25	0.9300
C3—C4	1.375 (7)	C26—C27	1.395 (8)
C3—C6	1.467 (7)	C26—H26	0.9300
C4—C5	1.375 (7)	C27—C28	1.353 (7)
C4—H4	0.9300	C27—C30	1.482 (8)
C5—H5	0.9300	C28—C29	1.392 (8)
C7—C8	1.464 (9)	C28—H28	0.9300
C7—H7A	0.9700	C29—H29	0.9300
C7—H7B	0.9700	C31—C32	1.488 (10)
C8—H8A	0.9600	C31—H31A	0.9700
C8—H8B	0.9600	C31—H31B	0.9700
C8—H8C	0.9600	C32—H32A	0.9600
C9—C10	1.369 (8)	C32—H32B	0.9600
C9—H9	0.9300	C32—H32C	0.9600
C10—C11	1.356 (9)		
			
N6—Co1—N5	177.8 (2)	O3—C14—C11	112.5 (8)
N6—Co1—N3	90.12 (17)	O3—C15—C16	105 (3)
N5—Co1—N3	87.69 (17)	O3—C15—H15A	110.8
N6—Co1—N2	90.37 (18)	C16—C15—H15A	110.8
N5—Co1—N2	90.09 (18)	O3—C15—H15B	110.8
N3—Co1—N2	91.72 (16)	C16—C15—H15B	110.8
N6—Co1—N1	91.34 (17)	H15A—C15—H15B	108.9
N5—Co1—N1	90.87 (18)	O3—C15'—C16'	109 (8)
N3—Co1—N1	175.75 (16)	O3—C15'—H15C	109.8
N2—Co1—N1	84.29 (16)	C16'—C15'—H15C	109.8
N6—Co1—N4	89.01 (17)	O3—C15'—H15D	109.8
N5—Co1—N4	90.62 (18)	C16'—C15'—H15D	109.8
N3—Co1—N4	90.65 (16)	H15C—C15'—H15D	108.2
N2—Co1—N4	177.55 (18)	C15'—C16'—H16D	109.5
N1—Co1—N4	93.36 (16)	C15'—C16'—H16E	109.5
C1—N1—C5	116.8 (5)	H16D—C16'—H16E	109.5
C1—N1—Co1	122.3 (4)	C15'—C16'—H16F	109.5
C5—N1—Co1	118.6 (4)	H16D—C16'—H16F	109.5
C13—N2—C9	117.7 (5)	H16E—C16'—H16F	109.5
C13—N2—Co1	123.0 (4)	N3—C17—C18	122.4 (5)
C9—N2—Co1	119.0 (4)	N3—C17—H17	118.8
C17—N3—C21	117.4 (5)	C18—C17—H17	118.8
C17—N3—Co1	120.3 (3)	C19—C18—C17	118.8 (5)
C21—N3—Co1	122.1 (4)	C19—C18—H18	120.6
C29—N4—C25	116.6 (5)	C17—C18—H18	120.6
C29—N4—Co1	120.8 (4)	C20—C19—C18	118.6 (5)
C25—N4—Co1	122.5 (4)	C20—C19—C22	119.3 (5)
C33—N5—Co1	162.0 (5)	C18—C19—C22	122.1 (6)
C34—N6—Co1	160.2 (5)	C21—C20—C19	119.5 (5)
C6—O1—C7	116.8 (5)	C21—C20—H20	120.3
C14—O3—C15'	121 (2)	C19—C20—H20	120.3
C14—O3—C15	110.5 (13)	N3—C21—C20	123.2 (5)
C15'—O3—C15	42 (2)	N3—C21—H21	118.4
C22—O5—C23	117.6 (5)	C20—C21—H21	118.4
C30—O7—C31	118.0 (6)	O6—C22—O5	124.5 (6)
N1—C1—C2	123.8 (5)	O6—C22—C19	123.0 (6)
N1—C1—H1	118.1	O5—C22—C19	112.5 (6)
C2—C1—H1	118.1	C24—C23—O5	111.1 (7)
C3—C2—C1	118.3 (5)	C24—C23—H23A	109.4
C3—C2—H2	120.8	O5—C23—H23A	109.4
C1—C2—H2	120.8	C24—C23—H23B	109.4
C2—C3—C4	118.4 (5)	O5—C23—H23B	109.4
C2—C3—C6	123.0 (5)	H23A—C23—H23B	108.0
C4—C3—C6	118.6 (5)	C23—C24—H24A	109.5
C3—C4—C5	119.0 (5)	C23—C24—H24B	109.5
C3—C4—H4	120.5	H24A—C24—H24B	109.5
C5—C4—H4	120.5	C23—C24—H24C	109.5
N1—C5—C4	123.6 (5)	H24A—C24—H24C	109.5
N1—C5—H5	118.2	H24B—C24—H24C	109.5
C4—C5—H5	118.2	N4—C25—C26	123.7 (6)
O2—C6—O1	123.3 (5)	N4—C25—H25	118.2
O2—C6—C3	123.1 (6)	C26—C25—H25	118.2
O1—C6—C3	113.6 (5)	C25—C26—C27	119.1 (5)
O1—C7—C8	112.0 (6)	C25—C26—H26	120.5
O1—C7—H7A	109.2	C27—C26—H26	120.5
C8—C7—H7A	109.2	C28—C27—C26	117.2 (5)
O1—C7—H7B	109.2	C28—C27—C30	122.3 (6)
C8—C7—H7B	109.2	C26—C27—C30	120.5 (6)
H7A—C7—H7B	107.9	C27—C28—C29	119.8 (6)
C7—C8—H8A	109.5	C27—C28—H28	120.1
C7—C8—H8B	109.5	C29—C28—H28	120.1
H8A—C8—H8B	109.5	N4—C29—C28	123.6 (6)
C7—C8—H8C	109.5	N4—C29—H29	118.2
H8A—C8—H8C	109.5	C28—C29—H29	118.2
H8B—C8—H8C	109.5	O8—C30—O7	125.1 (6)
N2—C9—C10	122.5 (6)	O8—C30—C27	121.9 (7)
N2—C9—H9	118.8	O7—C30—C27	113.0 (6)
C10—C9—H9	118.8	O7—C31—C32	106.3 (6)
C11—C10—C9	119.6 (6)	O7—C31—H31A	110.5
C11—C10—H10	120.2	C32—C31—H31A	110.5
C9—C10—H10	120.2	O7—C31—H31B	110.5
C10—C11—C12	119.5 (6)	C32—C31—H31B	110.5
C10—C11—C14	117.0 (7)	H31A—C31—H31B	108.7
C12—C11—C14	123.5 (7)	C31—C32—H32A	109.5
C11—C12—C13	117.7 (6)	C31—C32—H32B	109.5
C11—C12—H12	121.1	H32A—C32—H32B	109.5
C13—C12—H12	121.1	C31—C32—H32C	109.5
N2—C13—C12	123.0 (6)	H32A—C32—H32C	109.5
N2—C13—H13	118.5	H32B—C32—H32C	109.5
C12—C13—H13	118.5	N5—C33—S1	179.1 (6)
O4—C14—O3	125.6 (9)	N6—C34—S2	176.5 (6)
O4—C14—C11	121.8 (9)		
			
N6—Co1—N1—C1	161.4 (4)	C4—C3—C6—O2	−8.7 (9)
N5—Co1—N1—C1	−18.4 (4)	C2—C3—C6—O1	−8.2 (8)
N3—Co1—N1—C1	−89 (3)	C4—C3—C6—O1	174.0 (5)
N2—Co1—N1—C1	−108.4 (4)	C6—O1—C7—C8	−82.6 (7)
N4—Co1—N1—C1	72.3 (4)	C13—N2—C9—C10	−2.5 (9)
N6—Co1—N1—C5	−36.5 (4)	Co1—N2—C9—C10	−176.9 (5)
N5—Co1—N1—C5	143.8 (4)	N2—C9—C10—C11	2.0 (10)
N3—Co1—N1—C5	74 (3)	C9—C10—C11—C12	−0.3 (11)
N2—Co1—N1—C5	53.7 (4)	C9—C10—C11—C14	−177.0 (7)
N4—Co1—N1—C5	−125.6 (4)	C10—C11—C12—C13	−0.7 (10)
N6—Co1—N2—C13	−25.3 (5)	C14—C11—C12—C13	175.8 (6)
N5—Co1—N2—C13	152.5 (5)	C9—N2—C13—C12	1.4 (9)
N3—Co1—N2—C13	64.8 (5)	Co1—N2—C13—C12	175.6 (4)
N1—Co1—N2—C13	−116.7 (5)	C11—C12—C13—N2	0.1 (9)
N4—Co1—N2—C13	−101 (5)	C15'—O3—C14—O4	−32 (4)
N6—Co1—N2—C9	148.8 (4)	C15—O3—C14—O4	14 (2)
N5—Co1—N2—C9	−33.4 (5)	C15'—O3—C14—C11	144 (4)
N3—Co1—N2—C9	−121.1 (4)	C15—O3—C14—C11	−169.8 (15)
N1—Co1—N2—C9	57.5 (4)	C10—C11—C14—O4	2.2 (13)
N4—Co1—N2—C9	73 (5)	C12—C11—C14—O4	−174.3 (9)
N6—Co1—N3—C17	−47.3 (4)	C10—C11—C14—O3	−173.7 (8)
N5—Co1—N3—C17	132.3 (4)	C12—C11—C14—O3	9.7 (12)
N2—Co1—N3—C17	−137.7 (4)	C14—O3—C15—C16	−137.8 (17)
N1—Co1—N3—C17	−157 (2)	C15'—O3—C15—C16	−24 (3)
N4—Co1—N3—C17	41.7 (4)	C14—O3—C15'—C16'	96 (4)
N6—Co1—N3—C21	138.5 (4)	C15—O3—C15'—C16'	9 (3)
N5—Co1—N3—C21	−41.9 (4)	C21—N3—C17—C18	3.6 (9)
N2—Co1—N3—C21	48.1 (4)	Co1—N3—C17—C18	−170.9 (5)
N1—Co1—N3—C21	28 (3)	N3—C17—C18—C19	−1.9 (9)
N4—Co1—N3—C21	−132.5 (4)	C17—C18—C19—C20	−1.4 (9)
N6—Co1—N4—C29	154.9 (4)	C17—C18—C19—C22	177.4 (6)
N5—Co1—N4—C29	−22.9 (4)	C18—C19—C20—C21	2.9 (9)
N3—Co1—N4—C29	64.8 (4)	C22—C19—C20—C21	−175.9 (6)
N2—Co1—N4—C29	−130 (4)	C17—N3—C21—C20	−2.0 (9)
N1—Co1—N4—C29	−113.8 (4)	Co1—N3—C21—C20	172.4 (5)
N6—Co1—N4—C25	−21.5 (4)	C19—C20—C21—N3	−1.2 (9)
N5—Co1—N4—C25	160.7 (4)	C23—O5—C22—O6	1.3 (11)
N3—Co1—N4—C25	−111.6 (4)	C23—O5—C22—C19	−178.2 (6)
N2—Co1—N4—C25	54 (5)	C20—C19—C22—O6	−7.1 (10)
N1—Co1—N4—C25	69.8 (4)	C18—C19—C22—O6	174.2 (7)
N6—Co1—N5—C33	15 (6)	C20—C19—C22—O5	172.5 (6)
N3—Co1—N5—C33	4.7 (15)	C18—C19—C22—O5	−6.2 (9)
N2—Co1—N5—C33	−87.1 (15)	C22—O5—C23—C24	87.8 (9)
N1—Co1—N5—C33	−171.3 (15)	C29—N4—C25—C26	−0.2 (8)
N4—Co1—N5—C33	95.3 (15)	Co1—N4—C25—C26	176.4 (4)
N5—Co1—N6—C34	155 (5)	N4—C25—C26—C27	−1.1 (9)
N3—Co1—N6—C34	165.0 (14)	C25—C26—C27—C28	2.3 (8)
N2—Co1—N6—C34	−103.3 (14)	C25—C26—C27—C30	−177.7 (5)
N1—Co1—N6—C34	−19.0 (14)	C26—C27—C28—C29	−2.4 (8)
N4—Co1—N6—C34	74.3 (14)	C30—C27—C28—C29	177.7 (5)
C5—N1—C1—C2	−2.0 (8)	C25—N4—C29—C28	0.2 (8)
Co1—N1—C1—C2	160.5 (4)	Co1—N4—C29—C28	−176.4 (4)
N1—C1—C2—C3	−0.5 (8)	C27—C28—C29—N4	1.1 (9)
C1—C2—C3—C4	3.3 (8)	C31—O7—C30—O8	1.2 (10)
C1—C2—C3—C6	−174.5 (5)	C31—O7—C30—C27	−178.2 (5)
C2—C3—C4—C5	−3.7 (8)	C28—C27—C30—O8	−170.3 (6)
C6—C3—C4—C5	174.3 (5)	C26—C27—C30—O8	9.7 (9)
C1—N1—C5—C4	1.6 (8)	C28—C27—C30—O7	9.1 (8)
Co1—N1—C5—C4	−161.5 (4)	C26—C27—C30—O7	−170.9 (5)
C3—C4—C5—N1	1.2 (9)	C30—O7—C31—C32	175.2 (6)
C7—O1—C6—O2	2.4 (9)	Co1—N5—C33—S1	−101 (39)
C7—O1—C6—C3	179.7 (5)	Co1—N6—C34—S2	27 (11)
C2—C3—C6—O2	169.1 (6)		
